# Comparative effects of *Myrmecodia* sp. extract and infusion on organ function biomarkers, lipid metabolism, and meat lipid profile in avian pathogenic *Escherichia coli*-infected broiler chickens: Implications for sustainable poultry production within a One Health framework

**DOI:** 10.14202/vetworld.2026.1654-1664

**Published:** 2026-04-25

**Authors:** Ertika Fitri Lisnanti, Widya Paramita Lokapirnasari, Mohammad Anam Al Arif, Eka Pramyrtha Hestianah, Aswin Rafif Khairullah, Iwan Sahrial Hamid, Wiwiek Tyasningsih, Mirni Lamid, Mudhita Zikkrullah Ritonga, Farida Subasnia Dwijayanti, Amiril Mukmin, Saifur Rehman, Viski Fitri Hendrawan, Miarsono Sigit

**Affiliations:** 1 Doctoral Program of Veterinary Science, Faculty of Veterinary Medicine, Universitas Airlangga, Jl. Dr. Ir. H. Soekarno, Kampus C Mulyorejo, Surabaya 60115, East Java, Indonesia; 2 Program of Animal Husbandry, Faculty of Agriculture, Universitas Islam Kadiri, Kediri. Jl. Sersan Suharmaji 38, Kediri 64128, East Java, Indonesia; 3 Department of Animal Husbandry, Faculty of Veterinary Medicine, Universitas Airlangga, Jl. Mulyorejo, Kampus C Mulyorejo, Surabaya 60115, East Java, Indonesia; 4 Department of Veterinary Anatomy, Faculty of Veterinary Medicine, Universitas Airlangga, Jl. Mulyorejo, Kampus C Mulyorejo, Surabaya 60115, East Java, Indonesia; 5 Research Center for Veterinary Science, National Research and Innovation Agency (BRIN), Jl. Raya Bogor Km. 46 Cibinong, Bogor 16911, West Java, Indonesia; 6 Department of Basic Veterinary Medicine, Faculty of Veterinary Medicine, Universitas Airlangga, Jl. Mulyorejo, Kampus C Mulyorejo, Surabaya 60115, East Java, Indonesia; 7 Department of Veterinary Microbiology, Faculty of Veterinary Medicine, Universitas Airlangga, Jl. Mulyorejo, Kampus C Mulyorejo, Surabaya 60115, East Java, Indonesia; 8 Laboratory of Anatomy, Faculty of Veterinary Medicine, Universitas Syiah Kuala, Jl. Teuku Nyak Arief No. 441, Kopelma Darussalam, Banda Aceh 23111, Aceh, Indonesia; 9 Department of Pathobiology, Faculty of Veterinary and Animal Sciences, Gomal University, RV9W+GVJ, Indus HWY, Dera Ismail Khan 27000, Pakistan; 10 Department of Veterinary Reproduction, Faculty of Veterinary Medicine, Universitas Brawijaya, Jl. Puncak Dieng, Kunci, Kalisongo, Dau, Malang 65151, East Java, Indonesia; 11 Faculty of Veterinary Medicine, Universitas Wijaya Kusuma Surabaya, Jl. Dukuh Kupang XXV No. 54, Dukuh Kupang, Dukuh Pakis, Surabaya 60225, East Java Indonesia

**Keywords:** APEC, broiler chickens, lipid metabolism, *Myrmecodia* sp, organ biomarkers, phytogenic additive, sustainable poultry production, One Health

## Abstract

**Background and Aim:**

Avian pathogenic *Escherichia coli* (APEC) is a major cause of colibacillosis in broiler chickens, leading to systemic inflammation, organ dysfunction, disrupted lipid metabolism, and compromised meat quality. Growing concerns about antimicrobial resistance necessitate the development of sustainable alternatives to antibiotic growth promoters. *Myrmecodia* sp., a medicinal plant rich in bioactive flavonoids, tannins, and phenolics, has demonstrated antibacterial and antioxidant properties. However, limited information is available on the comparative effects of different preparation forms on organ function and lipid metabolism under APEC challenge conditions. This study aimed to evaluate the effects of *Myrmecodia* sp. extract and infusion on liver and kidney biomarkers, serum lipid profile, and meat lipid composition in APEC-infected broiler chickens.

**Materials and Methods:**

A total of 56 male Lohmann MB 202 broiler chickens were randomly assigned to seven groups: negative control, positive APEC-infected control, antibiotic control (zinc bacitracin), two extract treatments (15% and 30%), and two infusion treatments (1% and 2%). Treatments were administered from day 8 to day 35, and APEC infection was induced orally on day 21. Measured parameters included serum glutamate pyruvate transaminase (SGPT), serum glutamate oxaloacetate transaminase (SGOT), blood urea nitrogen (BUN), creatinine, low-density lipoprotein (LDL), high-density lipoprotein (HDL), and meat LDL and HDL. Data were analyzed using one-way analysis of variance followed by Duncan’s multiple range test (p < 0.05).

**Results:**

APEC infection significantly increased SGPT, SGOT, BUN, creatinine, and LDL levels while decreasing HDL levels (*p* < 0.05). Supplementation with Myrmecodia sp. extract and infusion significantly improved all evaluated parameters compared with the positive control (p < 0.05). Among treatments, the 1% infusion consistently produced the most favorable effects, including reduced liver enzyme activity, improved renal function, decreased LDL levels, and increased HDL levels in both serum and meat.

**Conclusion:**

*Myrmecodia* sp., particularly in infusion form, demonstrated protective effects on organ function and lipid metabolism in broilers infected with APEC. These findings support its potential as a phytogenic alternative to antibiotics in sustainable poultry production systems within a One Health framework.

## INTRODUCTION

Avian pathogenic *Escherichia coli* (APEC) is the primary cause of colibacillosis in poultry and poses a significant challenge for the global poultry industry [1]. APEC infection can trigger systemic inflammation, organ damage, reduced growth performance, and increased mortality in broiler chickens [2]. These effects not only lead to economic losses from treatment costs and reduced carcass quality but also potentially threaten public health if APEC strains carry antibiotic resistance genes that can be transferred to zoonotic pathogens [3]. Within a One Health framework, APEC control must address animal health, food safety, environmental contamination, and the broader issue of antimicrobial resistance (AMR) [4].

For decades, antibiotics have been widely used as growth promoters and for treating infections in poultry [5]. However, growing concerns about AMR have spurred the development of safer, more effective, and sustainable natural alternatives [6]. Among natural resources, medicinal plants rich in bioactive compounds have attracted increasing attention as phytogenic feed additives. One such plant is *Myrmecodia* sp., locally known as sarang semut (ant nest), an epiphytic plant traditionally used in Indonesia and Papua New Guinea [7]. *Myrmecodia* sp. contains secondary metabolites, including flavonoids, tannins, saponins, and phenolic compounds, and has reported antibacterial, antioxidant, anti-inflammatory, and immunomodulatory activities [8]. Compared with other phytotherapeutic agents frequently investigated in poultry nutrition, *Myrmecodia* sp. has been less extensively studied in controlled APEC challenge models, particularly regarding its effects on biomarkers of organ function and lipid metabolism [9–11].

In cases of APEC infection, damage to organs such as the liver, kidneys, and intestines results from bacterial colonization and an excessive inflammatory response [12]. Disruption of these organs can impair lipid metabolism, reduce detoxification, and lower feed utilization efficiency [13]. APEC infection is also often accompanied by decreased meat quality, including changes in intramuscular fat content, color, pH, and water-holding capacity, ultimately reducing the market value of poultry products [14]. Therefore, interventions that maintain organ integrity, stabilize lipid metabolism, and preserve meat quality are necessary to support the production of healthy, high-quality broilers [3].

Preparation methods for *Myrmecodia* sp., including ethanolic extracts and aqueous infusions, may yield distinct phytochemical profiles due to differences in solvent polarity [15, 16]. Ethanol-based extracts are generally richer in flavonoids and phenolic compounds, whereas aqueous infusions may contain higher proportions of water-soluble constituents such as tannins and saponins. Understanding these preparation-dependent differences is essential for optimizing biological efficacy in poultry systems. Previous reports from the same experimental trial have described growth performance, immune responses, and phytochemical characteristics of *Myrmecodia* sp. supplementation under APEC challenge. However, a focused evaluation of biochemical liver and kidney markers, together with serum and meat lipid profiles, has not been comprehensively addressed [17]. The One Health perspective emphasizes that plant-based interventions may reduce antibiotic dependency while supporting animal health and food safety [18, 19].

Despite the growing body of research on phytogenic feed additives as alternatives to antibiotics in poultry production, significant gaps remain in understanding their organ-level and metabolic effects under infectious challenge conditions. Previous studies on *Myrmecodia* sp. have primarily focused on growth performance, immune modulation, and general antimicrobial activity, with limited emphasis on biochemical indicators of organ function. In particular, there is a lack of comprehensive evaluation of liver and kidney biomarkers, such as serum glutamate pyruvate transaminase (SGPT), serum glutamate oxaloacetate transaminase (SGOT), blood urea nitrogen (BUN), and creatinine, in broiler chickens exposed to APEC. Although plant-derived compounds have been reported to influence lipid metabolism in poultry [20], studies investigating the specific effects of *Myrmecodia* sp. on serum lipid fractions, including low-density lipoprotein (LDL) and high-density lipoprotein (HDL), as well as corresponding lipid composition in meat, remain scarce. Another important gap is the limited comparative assessment of different preparation methods of *Myrmecodia* sp., particularly ethanolic extracts versus aqueous infusions, which are known to yield distinct phytochemical profiles and may result in differential biological effects. In addition, most existing studies have not integrated these physiological and biochemical outcomes within a One Health framework, which is essential for addressing AMR and ensuring sustainable poultry production. Therefore, a systematic investigation linking organ function, lipid metabolism, and meat quality under APEC challenge conditions is critically needed.

Addressing these gaps, the present study aimed to comprehensively evaluate the effects of *Myrmecodia* sp. extract and infusion on key physiological and biochemical parameters in broiler chickens experimentally infected with APEC. Specifically, this study assessed the impact of different concentrations of *Myrmecodia* sp. extract and aqueous infusion on liver function biomarkers (SGPT and SGOT), kidney function biomarkers (BUN and creatinine), serum lipid profile (LDL and HDL), and meat lipid composition. Furthermore, this study compared the relative efficacy of extract and infusion preparations in mitigating APEC-induced physiological disturbances, thereby identifying the most effective formulation for improving animal health and product quality. By integrating these findings, the study also aimed to provide scientific evidence supporting the use of *Myrmecodia* sp. as a phytogenic alternative to antibiotic growth promoters, contributing to reduced antimicrobial use and aligning poultry production practices with the One Health approach.

## MATERIALS AND METHODS

### Ethical approval

The experimental protocol involving broiler chickens was reviewed and approved by the Institutional Ethical Committee of Universitas Airlangga, Indonesia, under approval number 1.KEH.005.01.2024. All animal procedures were conducted in accordance with institutional guidelines for the care and use of animals in research and complied with internationally accepted principles of animal welfare and humane experimentation. The study design, handling procedures, oral challenge model, sample collection, and endpoint assessments were planned to minimize animal stress, pain, and unnecessary suffering throughout the experimental period. Birds were maintained under appropriate husbandry conditions, including adequate housing, ventilation, temperature, lighting, and provision of feed and water, and were monitored daily for general health status and clinical signs following the APEC challenge. All interventions were performed by trained personnel using standard operating procedures. Blood collection was performed carefully at the end of the experimental period using appropriate restraint techniques to minimize distress. No procedures beyond those approved by the ethics committee were performed during the study.

### Study period and location

This study was conducted from January to August 2024. The research was carried out at the partner poultry farm of Universitas Islam Kadiri, located in Blabak, Pesantren District, Kediri City, East Java, Indonesia. Birds were housed in floor pens under standard commercial management conditions, with ad libitum access to feed and water. Temperature, ventilation, and lighting were maintained according to broiler management guidelines. Serum biochemical parameters were analyzed at OKTA SAINTIKA Laboratory in Malang, East Java, Indonesia. The APEC used in this study was an isolated and identified strain maintained at the Microbiology Laboratory, Faculty of Veterinary Medicine, Universitas Brawijaya.

### Experimental design

The material used in this study consisted of Lohmann strain (MB 202) broiler chickens, a commercial strain produced by PT Japfa Comfeed Indonesia. A total of 56 male broilers were allocated to seven treatment groups, with each treatment replicated four times and two birds per replicate (n = 8 birds/treatment). The number of birds per treatment was determined based on comparable experimental studies evaluating biochemical parameters in broilers under infectious challenge models, in which similar group sizes have been reported to be adequate for detecting statistically significant differences.

The treatment groups were arranged as follows:

K–: Negative control (uninfected, untreated)

K+: Positive control (APEC-infected only)

P0: APEC-infected + zinc bacitracin (Antibiotic growth promoter [AGP] control; 0.5 g/kg feed)

P1: APEC-infected + *Myrmecodia* sp. extract 15% at 0.1% concentration in drinking water

P2: APEC-infected + *Myrmecodia* sp. extract 30% at 0.1% concentration in drinking water

P3: APEC-infected + *Myrmecodia* sp. infusion at 1% of total drinking water

P4: APEC-infected + *Myrmecodia* sp. infusion at 2% of total drinking water

Extracts and infusions were administered daily from day 8 to day 35. APEC infection was performed on day 21 via oral administration of a 10^8^ CFU/mL bacterial suspension prepared in sterile saline [21].

### Preparation of *Myrmecodia* sp. extract

The tubers of the ant nest plant (*Myrmecodia* sp.) were cleaned, sliced, and dried in direct sunlight. The dried material was ground into a fine powder and macerated in 96% ethanol (Merck, Darmstadt, Germany) at a 1:10 (w/v) ratio for 24 h at 25°C. The mixture was filtered twice, and the filtrate was concentrated with a rotary evaporator (Heidolph, Schwabach, Germany) at 60°C to obtain a crude extract. The extract was stored at 4°C until further use [10]. The plant material was obtained from local collectors in Papua, Indonesia, and taxonomically authenticated before experimental use. The phytochemical profile of the *Myrmecodia* sp. batch used in this study, including flavonoids, phenolic compounds, tannins, and saponins, has been previously characterized and reported [22].

### Preparation of *Myrmecodia* sp. infusion

The infusion was prepared by boiling powdered *Myrmecodia* sp. in distilled water (Brataco, Jakarta, Indonesia). For the 1% infusion, 1 g of powdered simplicia was boiled in 100 mL of distilled water at 90°C for 15 min, then cooled and filtered through muslin cloth. For the 2% infusion, 2 g of powdered simplicia was processed using the same procedure with 100 mL of distilled water. All infusions were freshly prepared daily and administered via drinking water according to the respective treatment groups [22].

### Purification method for field isolate of *E. coli* O78

The field isolate, *E. coli* O78, was initially enriched in 0.1% Buffered Peptone Water (Oxoid, Hampshire, UK) and incubated at 37°C for 24 h, with turbidity indicating bacterial growth. The culture was then streaked onto Sorbitol MacConkey Agar (Oxoid, Hampshire, UK) and incubated under the same conditions. On Sorbitol MacConkey Agar, *E. coli* O78 colonies were round, nucleated, and colorless due to the strain’s inability to ferment sorbitol [23]. Colorless colonies were subcultured onto Eosin Methylene Blue Agar (Oxoid, Hampshire, UK) and incubated at 37°C for 24 h, where colonies with a characteristic green metallic sheen and dark centers confirmed *E. coli* growth [24].

Biochemical identification was further performed using Triple Sugar Iron Agar, Sulfide Indole Motility medium, Methyl Red–Voges Proskauer medium, citrate utilization medium, and urease medium (Oxoid, Hampshire, UK), confirming the isolate as *E. coli* O78. The confirmed isolate was then standardized to a 0.5 McFarland turbidity in sterile saline with a densitometer to prepare the inoculum for experimental infection [25].

### Sample collection and blood analysis

At the end of the experiment (day 35), blood samples were collected from the brachial vein of one bird per replicate using both ethylenediaminetetraacetic acid (EDTA) and plain vacutainer tubes (BD, Franklin Lakes, NJ, USA). The samples were centrifuged at 1,000 × *g* for 15 min, after which the sera were separated and stored at –20°C until biochemical analyses were performed [26].

Serum biochemical parameters were analyzed at OKTA SAINTIKA Laboratory, Malang, East Java, Indonesia, using standard commercial reagent kits. The following assays were conducted:

SGPT and SGOT were measured spectrophotometrically using the Humazym-UV Test (Human Diagnostics, Wiesbaden, Germany) at a wavelength of 340 nm. BUN was determined using the enzymatic UV method at a wavelength of 578 nm. Creatinine was measured using the alkaline picrate method at a wavelength of 520 nm. LDL and HDL were analyzed using the enzymatic CHOD–PAP method at a wavelength of 500 nm.

### Measurement of HDL and LDL cholesterol in meat

Cholesterol levels in meat, specifically HDL and LDL, were measured using the chloroform extraction method. About 4 g of minced meat was extracted with chloroform (Merck, Darmstadt, Germany) in three 25 mL portions. The lipid layer was then evaporated to dryness, and the remaining fat residue was transferred into a test tube, covered with aluminum foil to protect it from light, and analyzed enzymatically using a photometric method. Cholesterol was quantified after enzymatic hydrolysis and oxidation, which yielded quinoneimine from hydrogen peroxide and aminophenazone in the presence of phenol and peroxidase [27].

### Observation variables

The observed variables included serum levels of SGPT, SGOT, creatinine, BUN, LDL, and HDL, as well as LDL and HDL concentrations in meat.

### Statistical analysis

All data were analyzed using analysis of variance under a completely randomized design. When significant effects were observed (p < 0.05), mean comparisons were conducted using Duncan’s multiple range test at the 5% level. Statistical analyses were conducted using IBM SPSS Statistics version 26.0 (IBM Corp., NY, USA)

## RESULTS

### Liver function enzymes (SGPT and SGOT)

The mean SGOT and SGPT values in APEC-infected broilers treated with *Myrmecodia* sp. extract or infusion are presented in Table 1. Administration of *Myrmecodia* sp. extract or infusion significantly affected both SGPT and SGOT levels (p < 0.05).

**Table 1:** Average levels of SGPT and SGOT in broiler chickens.

Treatment	SGPT level (U/L)	SGOT level (U/L)
K–	68.04 ± 6.16^a^	78.41 ± 1.59^bc^
K+	157.60 ± 9.73^d^	141.71 ± 6.68^d^
P0	122.46 ± 5.15^bc^	128.45 ± 5.45^d^
P1	139.51 ± 28.52^cd^	85.16 ± 13.80^c^
P2	125.51 ± 18.41^bc^	67.69 ± 3.60^b^
P3	64.59 ± 4.45^a^	40.40 ± 16.02^a^
P4	104.61 ± 5.54^b^	75.81 ± 12.41^bc^

Administration of ant nest extract and herbal infusion of ant nests infected with *Escherichia coli* bacteria to broiler chickens showed a significant effect (*p* < 0.05) on SGPT and SGOT. SGPT = Serum glutamate pyruvate transaminase, SGOT = Serum glutamate oxaloacetate transaminase.

SGPT levels ranged from 64.59 to 157.60 U/L. The lowest value was observed in the negative control (K–) at 68.04 U/L, while the highest was in the positive APEC-infected control (K+) at 157.60 U/L. The AGP control (P0) also showed elevated SGPT levels (122.46 U/L). Treatments with *Myrmecodia* sp. extract reduced SGPT levels, particularly in P1 (15% extract; 139.51 U/L) and P2 (30% extract; 125.51 U/L). Infusion treatments showed more pronounced improvement, with P3 (1% infusion) recording the lowest SGPT value (64.59 U/L), followed by P4 (2% infusion; 104.61 U/L).

Similar trends were observed for SGOT activity. Compared with the AGP control (P0), both extract and infusion treatments reduced transaminase levels, indicating improved hepatic integrity. The 15% extract treatment (P1) also showed a measurable hepatoprotective effect.

### Kidney function parameters (BUN and creatinine)

The average BUN and creatinine levels of APEC-infected broilers treated with *Myrmecodia* sp. are shown in Table 2. Treatments significantly affected BUN levels (p < 0.05), which ranged from 6.11 to 25.48 mmol/L. The lowest BUN level was observed in K– (8.97 mmol/L), while K+ showed the highest value (25.48 mmol/L). The AGP control (P0) recorded 9.56 mmol/L. Among the treatments, P3 (1% infusion) showed the greatest reduction in BUN, indicating improved renal function.

**Table 2:** Average levels of blood urea nitrogen and creatinine in broiler chickens.

Treatment	BUN level (mmol/L)	Creatinine level (U/L)
K–	8.97 ± 0.82^a^	0.53 ± 0.19^a^
K+	25.48 ± 5.12^d^	4.66 ± 0.37^d^
P0	9.56 ± 0.96^ab^	2.94 ± 0.83^c^
P1	16.68 ± 4.19^c^	2.67 ± 1.22^c^
P2	17.12 ± 7.31^c^	1.46 ± 0.71^ab^
P3	6.11 ± 1.57^a^	2.05 ± 0.42^bc^
P4	15.82 ± 5.62^bc^	1.98 ± 0.27^bc^

Administration of ant nest extract and herbal infusion of ant nest infected with *Escherichia coli* bacteria to broiler chickens showed that it had a significant effect (p < 0.05) on BUN and creatinine levels. BUN = Blood urea nitrogen.

Extract treatments resulted in BUN values of 16.68 mmol/L (P1) and 17.12 mmol/L (P2). Infusion treatments showed lower BUN levels, with P3 (1% infusion) at 6.11 mmol/L and P4 (2% infusion) at 15.82 mmol/L.

Creatinine levels also differed significantly among treatments (p < 0.05), ranging from 0.53 to 4.66 U/L. The lowest creatinine value was found in K– (0.53 U/L), while the highest occurred in K+ (4.66 U/L). The AGP control (P0) recorded 2.94 U/L. Extract treatments resulted in creatinine levels of 2.67 U/L (P1) and 1.46 U/L (P2). Infusion treatments yielded values of 2.05 U/L (P3) and 1.98 U/L (P4), indicating that infusion-based administration generally improved renal function. Overall, treated groups exhibited lower creatinine levels than the positive control group.

### Lipid profile (LDL and HDL) in serum

The effects of *Myrmecodia* sp. extract and infusion on serum LDL and HDL levels are summarized in Table 3. Both parameters were significantly affected by treatment (p < 0.05). Serum LDL levels ranged from 39.65 to 99.65 mg/dL. K– (55.85 mg/dL) had lower LDL values than K+ (99.55 mg/dL), while the AGP control (P0) recorded 79.17 mg/dL. Extract treatments showed LDL levels of 45.24 mg/dL (P1) and 43.52 mg/dL (P2). Infusion treatments produced the lowest LDL value in P3 (39.65 mg/dL), followed by P4 (46.62 mg/dL).

**Table 3:** Average serum levels of LDL and HDL.

Treatment	LDL (mg/dL)	HDL (mg/dL)
K–	55.85 ± 1.25^b^	64.88 ± 3.82^c^
K+	99.55 ± 10.38^d^	22.13 ± 5.32^a^
P0	79.17 ± 6.70^c^	71.78 ± 4.35^c^
P1	45.24 ± 5.42^a^	28.58 ± 11.62^a^
P2	43.52 ± 2.64^a^	26.94 ± 6.38^a^
P3	39.65 ± 1.83^a^	68.26 ± 10.16^c^
P4	46.62 ± 5.47^a^	50.11 ± 5.62^b^

Administration of ant nest extract and herbal infusion of ant nests infected with avian pathogenic *Escherichia coli* to broiler chickens showed a significant effect (p < 0.05) on serum LDL and HDL levels. LDL = Low density lipoprotein, HDL = High density lipoprotein.

Serum HDL levels ranged from 22.13 to 71.78 mg/dL. K– had the highest HDL level (64.88 mg/dL), whereas K+ had the lowest (22.13 mg/dL). The AGP control (P0) recorded 71.78 mg/dL. Extract treatments resulted in HDL values of 28.58 mg/dL (P1) and 26.94 mg/dL (P2). Infusion treatments showed HDL levels of 68.26 mg/dL (P3) and 50.11 mg/dL (P4). The best overall lipid modulation was observed in P3 (1% infusion), which produced the lowest LDL and one of the highest HDL levels among treatment groups.

### Lipid profile and cholesterol modulation in broiler meat

The effects of *Myrmecodia* sp. extract and infusion on meat LDL-C and HDL-C levels are summarized in Table 4. *Myrmecodia* sp. supplementation significantly improved both serum and meat lipid profiles in APEC-infected broilers (p < 0.05). Significant differences in meat LDL-C and HDL-C concentrations were observed among treatment groups (p < 0.05).

**Table 4:** Average meat levels of LDL and HDL.

Treatment	Meat LDL-C (mg/dL)	Meat HDL-C (mg/dL)
K–	55.03 ± 7.29^a^	60.68 ± 4.95^e^
K+	74.88 ± 1.07^bc^	27.80 ± 5.47^bc^
P0	79.47 ± 19.76^c^	15.52 ± 6.03^a^
P1	78.46 ± 12.17^c^	18.31 ± 6.74^ab^
P2	60.76 ± 6.41^ab^	32.47 ± 12.76^c^
P3	50.01 ± 9.53^a^	56.10 ± 7.74^de^
1	59.44 ± 8.68^ab^	45.62 ± 8.74^de^

Administration of ant nest extract and herbal infusion of ant nests infected with avian pathogenic *Escherichia coli* to broiler chickens showed a significant effect (*p* < 0.05) on serum LDL and HDL levels. LDL = Low density lipoprotein, HDL = High density lipoprotein.

Meat LDL-C levels ranged from 50.01 mg/dL (P3) to 79.47 mg/dL (P0). The positive control (K+) recorded 74.88 mg/dL, while the negative control (K–) showed 55.03 mg/dL. Lower meat LDL-C values were observed in P2 (60.76 mg/dL), P3 (50.01 mg/dL), and P4 (59.44 mg/dL) compared with the positive control.

Meat HDL-C levels ranged from 15.52 ± 6.03 mg/dL (P0) to 60.68 ± 4.95 mg/dL (K–). The positive control (K+) recorded 27.80 ± 5.47 mg/dL. Among treatment groups, P3 (1% infusion) showed 56.10 ± 7.74 mg/dL, while P4 recorded 45.62 ± 8.74 mg/dL.

## DISCUSSION

### Hepatoprotective effects of *Myrmecodia* sp. in APEC-induced liver dysfunction

The results of this study demonstrate that *Myrmecodia* sp. extract and infusion were associated with improvements in broilers infected with APEC, as reflected in enhanced liver and kidney function, lipid metabolism, and meat lipid composition. Importantly, this study extends previous findings on growth and immune responses by providing biochemical evidence of organ-level recovery under conditions of infectious stress. These findings support the growing interest in phytogenic compounds as potential complementary strategies to reduce antibiotic dependence, thereby supporting efforts to reduce AMR within a One Health framework.

APEC infection caused a substantial increase in SGPT and SGOT levels, indicating hepatocellular damage from systemic inflammation and endotoxin exposure [3]. The significant increase in transaminases in the positive control group aligns with the well-documented pathogenesis of colibacillosis, in which oxidative stress, lipopolysaccharide-induced inflammation, and impaired hepatocyte membrane integrity contribute to liver dysfunction [13].

Administration of *Myrmecodia* sp., particularly as an infusion (P3), was associated with lower SGPT and SGOT activities than the positive control. These findings suggest a potential hepatoprotective role, possibly attributable to flavonoids, phenolic compounds, and tannins, which modulate inflammatory pathways [10]. The infusion group showed lower transaminase values, which may reflect differences in phytochemical solubility. Water-soluble compounds such as tannins and saponins have been reported to enhance antioxidant enzyme activity and reduce lipid peroxidation, thereby limiting hepatocellular leakage under acute infectious stress [28, 29]. These observations indicate that preparation-dependent phytochemical availability may influence the magnitude of hepatic protection during systemic bacterial challenge.

### Renoprotective function and improvement of kidney biomarkers

APEC infection similarly impaired renal function, as indicated by elevated BUN and creatinine levels in the positive control. These increases reflect reduced glomerular filtration, renal inflammation, and nephron stress caused by systemic bacterial infection [30].

*Myrmecodia* sp. supplementation was associated with reduced levels of renal biomarkers compared with the positive control, particularly in the 1% infusion group (P3). The reduction in BUN and creatinine suggests improved glomerular filtration and reduced inflammation-mediated renal damage. Flavonoids and phenolic antioxidants may contribute to preserving renal microvascular integrity and mitigating oxidative stress-induced tubular injury, which are commonly observed in systemic infections. These findings provide biochemical support for a renoprotective effect under infectious conditions [31].

### Modulation of serum lipid metabolism

APEC infection disrupted lipid homeostasis, characterized by increased LDL and decreased HDL levels. These alterations align with previous reports indicating that systemic inflammation impairs hepatic lipid regulation, promotes lipid peroxidation, and accelerates HDL particle degradation [32].

Both extract and infusion treatments improved the serum lipid profile relative to the positive control. Notably, the 1% infusion group exhibited lower LDL and higher HDL concentrations compared with infected controls. This enhancement in lipid metabolism is likely mediated through the combined antioxidant and anti-inflammatory actions of *Myrmecodia* sp., which support hepatic recovery and optimize lipid processing [33]. Flavonoids and tannins may also modulate key lipid regulatory enzymes, potentially reducing LDL synthesis and enhancing HDL stability [34]. Phytogenic treatments demonstrated comparable or favorable effects on lipid parameters compared with zinc bacitracin (P0), suggesting potential metabolic modulation beyond antimicrobial activity [35]. The relatively elevated HDL concentration observed in the zinc bacitracin group may reflect antibiotic-associated modulation of lipid metabolism, possibly through alterations in gut microbiota composition and bile acid metabolism, thereby influencing hepatic lipoprotein regulation. However, this effect requires further investigation to clarify its underlying mechanisms.

### Improvements in meat lipid composition and implications for meat quality

APEC infection negatively influenced meat lipid composition. Treatment with *Myrmecodia* sp. was associated with higher HDL-C and lower LDL-C in meat compared with infected controls. Treatment with *Myrmecodia* sp. effectively counteracted these changes. The 1% infusion group exhibited HDL-C values approaching those of the negative control. This indicates improved intramuscular lipid metabolism and reduced oxidative stress within muscle tissues [10].

Improved lipid profiles in meat may reflect systemic metabolic stabilization and reduced oxidative stress in muscle tissues [36]. Enhanced meat lipid profiles are particularly important from the perspectives of food safety and consumer health [37]. Higher HDL and lower LDL levels contribute to better nutritional quality of poultry products, supporting public health goals and improving marketability [38]. By linking biochemical organ recovery to measurable changes in meat lipid composition, this study provides an integrative perspective that links animal physiology to product quality, a connection that has been less emphasized in previous *Myrmecodia*-based studies. These findings suggest that phytogenic supplementation may contribute to both animal health and product quality [39].

### Comparative performance of extract versus infusion

Across multiple evaluated parameters, infusion treatments, particularly P3 (1% infusion), demonstrated consistently favorable outcomes compared with extract treatments. Ethanol-based extraction typically concentrates specific flavonoids and phenolic compounds, whereas aqueous infusion yields a phytochemical profile enriched in water-soluble constituents. The observed differences in biological responses are consistent with variations in phytochemical solubility and bioavailability, particularly the presence of tannins and saponins in aqueous preparations [40]. These results highlight that the preparation method is not merely a technical variable but a biologically relevant factor influencing therapeutic efficacy under infectious stress. These findings emphasize that preparation methods play a critical role in shaping physiological responses and optimizing the therapeutic potential of phytogenic compounds in poultry systems [41].

### One Health implications and relevance to AMR mitigation

The findings of this study highlight the potential role of *Myrmecodia* sp. supplementation as a supportive strategy in broiler production systems aimated to reducing reliance on antibiotic growth promoters. The observed improvements in organ function biomarkers and lipid parameters indicate that phytogenic supplementation may help maintain physiological stability under infectious challenge conditions. Such strategies could contribute to reducing routine antibiotic use and, consequently, lowering selective pressure associated with AMR [35].

Within the One Health framework, these outcomes are aligned with international efforts to safeguard animal health while promoting food safety and responsible antimicrobial stewardship across animal, human, and environmental interfaces [42]. The use of locally available medicinal plants may further support sustainable livestock management practices and reduce dependence on synthetic pharmaceutical inputs [43].

## CONCLUSION

The present study demonstrated that supplementation with *Myrmecodia* sp. significantly mitigated the adverse physiological effects induced by APEC infection in broiler chickens. APEC challenge resulted in marked elevations in SGPT, SGOT, BUN, creatinine, and LDL levels, accompanied by a reduction in HDL levels, indicating hepatic dysfunction, renal impairment, and disrupted lipid metabolism. Administration of *Myrmecodia* sp., particularly in the 1% infusion form, effectively restored these parameters toward normal physiological ranges. The infusion treatment showed the lowest SGPT (64.59 U/L), SGOT (40.40 U/L), BUN (6.11 mmol/L), and LDL levels (39.65 mg/dL), along with improved HDL concentrations in both serum and meat, indicating superior hepatoprotective, renoprotective, and hypolipidemic effects compared with extract and antibiotic treatments.

From a practical perspective, these findings suggest that *Myrmecodia* sp., especially as an aqueous infusion, can serve as an effective phytogenic feed additive to enhance organ function, stabilize lipid metabolism, and improve meat lipid quality in broiler production systems under infectious stress. The observed improvements in meat HDL and LDL profiles further highlight its potential to enhance the nutritional quality and market value of poultry products, thereby supporting both animal health and consumer health within a One Health framework.

The major strength of this study lies in its comprehensive evaluation of organ function biomarkers, serum lipid profile, and meat lipid composition under a controlled APEC challenge model, and in its direct comparison of two preparation methods for *Myrmecodia* sp. (extract vs. infusion). This integrative approach provides novel insights into the physiological and metabolic responses to phytogenic supplementation and bridges the gap between animal health and product quality.

However, certain limitations should be acknowledged. The study was conducted under controlled experimental conditions, with a relatively small sample size and a single APEC strain, which may not fully reflect field variability. In addition, detailed mechanistic investigations at the molecular level, including gene expression and modulation of the gut microbiota, were not performed. The phytochemical composition was inferred from previous reports rather than quantified in the current experimental batch.

Future research should focus on large-scale field trials, dose optimization, and long-term safety evaluation of *Myrmecodia* sp. supplementation. Further studies are also needed to elucidate the molecular mechanisms underlying its hepatoprotective and lipid-modulating effects, including its interactions with genes, the gut microbiota, immune pathways, and lipids involved in lipid metabolism. Comparative studies with other phytogenic additives and economic feasibility assessments would further strengthen its application in commercial poultry production.

In conclusion, *Myrmecodia* sp., particularly in infusion form, represents a promising natural alternative to antibiotic growth promoters, capable of improving organ function, regulating lipid metabolism, and enhancing meat quality in broilers challenged with APEC. Its application aligns with sustainable poultry production strategies and contributes to AMR mitigation within the One Health paradigm.

## DATA AVAILABILITY

The supplementary data can be available from the corresponding author upon reasonable request.

## AUTHORS’ CONTRIBUTIONS

EFL and WPL: Conception and design of the study. MAA and EPH: Conducted the study and analyzed the results. ISH, and WT: Interpreted the results. MZR and FSD: Analysis and interpreted the data. ARK, FA, and SR: Study design, data interpretation, and drafted the manuscript. AM, MS, and ML: Methodology development, supervision, and critical revision of the manuscript. All authors have read and approved the final version of the manuscript.
